# Systemic hemostatic agents initiated in trauma patients in the pre-hospital setting: a systematic review

**DOI:** 10.1007/s00068-022-02185-6

**Published:** 2022-12-16

**Authors:** Annalisa Biffi, Gloria Porcu, Greta Castellini, Antonello Napoletano, Daniela Coclite, Daniela D’Angelo, Alice Josephine Fauci, Laura Iacorossi, Roberto Latina, Katia Salomone, Primiano Iannone, Silvia Gianola, Osvaldo Chiara, the Italian National Institute of Health Guideline Working Group, Alessia Medici, Alessandro Mazzola, Carlo Coniglio, Elvio De Blasio, Gaddo Flego, Massimo Geraci, Giulio Maccauro, Antonio Rampoldi, Federico Santolini, Claudio Tacconi, Gregorio Tugnoli, Nino Stocchetti, Andrea Fabbri, Maria Pia Ruggeri, Maurella Della Seta, Scilla Pizzarelli, Rosaria Rosanna Cammarano

**Affiliations:** 1 Department of Statistics and Quantitative Methods, National Centre for Healthcare Research and Pharmacoepidemiology, University of Milano-Bicocca, Milan, Italy; 2 Unit of Biostatistics, Epidemiology, and Public Health, Department of Statistics and Quantitative Methods, University of Milano-Bicocca, Milan, Italy; 3 Unit of Clinical Epidemiology, IRCCS Istituto Ortopedico Galeazzi, Milan, Italy; 4 Istituto Superiore di Sanità, Centro Eccellenza Clinica, Qualità e Sicurezza delle Cure, Rome, Italy; 5 Department of Pathophysiology and Transplantation, University of Milan, Milan, Italy; 6 General Surgery and Trauma Team, ASST Grande Ospedale Metropolitano Niguarda, University of Milan, Milan, Italy

**Keywords:** Systematic review, Major trauma, Pre-hospital, Systemic hemostatic agents

## Abstract

**Purpose:**

The effect of systemic hemostatic agents initiated during pre-hospital care of severely injured patients with ongoing bleeding or traumatic brain injury (TBI) remains controversial. A systematic review and meta-analysis was therefore conducted to assess the effectiveness and safety of systemic hemostatic agents as an adjunctive therapy in people with major trauma and hemorrhage or TBI in the context of developing the Italian National Institute of Health guidelines on major trauma integrated management.

**Methods:**

PubMed, Embase, and Cochrane Library databases were searched up to October 2021 for studies that investigated pre-hospital initiated treatment with systemic hemostatic agents. The certainty of evidence was evaluated with the Grading of Recommendations Assessment, Development, and Evaluation approach, and the quality of each study was determined with the Cochrane risk-of-bias tool. The primary outcome was overall mortality, and secondary outcomes included cause-specific mortality, health-related quality of life, any adverse effects and blood product use, hemorrhage expansion, and patient-reported outcomes.

**Results:**

Five trials of tranexamic acid (TXA) met the inclusion criteria for this meta-analysis. With a high certainty of evidence, when compared to placebo TXA reduced mortality at 24 h (relative risk = 0.83, 95% confidence interval = 0.73–0.94) and at 1 month among trauma patients (0.91, 0.85–0.97). These results depend on the subgroup of patients with significant hemorrhage because in the subgroup of TBI there are no difference between TXA and placebo. TXA also reduced bleeding death and multiple organ failure whereas no difference in health-related quality of life.

**Conclusion:**

Balancing benefits and harms, TXA initiated in the pre-hospital setting can be used for patients experiencing major trauma with significant hemorrhage since it reduces the risk of mortality at 24 h and one month with no difference in terms of adverse effects when compared to placebo. Considering the subgroup of severe TBI, no difference in mortality rate was found at 24 h and one month. These results highlight the need to conduct future studies to investigate the role of other systemic hemostatic agents in the pre-hospital settings.

## Introduction

Severe trauma is a major global public health issue [1]. According to the World Health Organization, the three most common causes of injury and violence-related deaths specifically road traffic accidents, suicides, and homicides. Furthermore, road traffic crashes and falls are the main causes of traumatic brain injury (TBI) [2]; intracranial bleeding is a possible complication of TBI [3], whose frequency depends on the injury severity [4]. Severely injured trauma patients are characterized by coagulation abnormalities and a substantially increased mortality rate [5]. Approximately 33% patients with TBI present with coagulopathy, which can increase the hemorrhage expansion and risk of death [4]. Hemorrhagic death generally occurs within the first few hours of injury (median time to hemorrhagic death is 2–2.6 h [6]), while late mortality is defined as death due to multiple organ failure or infection [7]. Over the last decade, increased awareness and improved pre-hospital care may have triggered a reduction in the number of severely injured patients with ongoing bleeding being transferred to a trauma center [8, 9]. Early detection and management of traumatic hemorrhage and TBI with intracranial hematoma can improve clinical outcomes [10], and, as suggested by the European guidelines, “current guidelines are needed for the implementation of evidence-based local protocols and algorithms together with parameters to assess key measures of bleeding control and outcome” [1, 9].

In trauma patients, interventions for bleeding may be required during the (1) acute phase (0–6 h from injury), with blood loss and shock; (2) intermediate phase (6–24 h from injury), with an increased risk of death due to severe TBI and concomitant physiologic impairment; and the (3) late phase, with the occurrence of inflammatory damages and/or complications [7]. Temporary measures to control hemorrhage in pre-hospital settings may be applied, such as the use of tourniquets, hemostatic dressings and pelvic binders, until definitive care is available. In non-compressible hemorrhage recent evidence suggests the resuscitative endovascular balloon occlusion of the aorta that however their use is still anedoctal [11]. Moreover, the use of pro-hemostatic agents has been suggested as an adjunctive measure to reduce bleeding and prevent trauma-induced coagulopathy [12] and to decrease the size of intracranial hematoma [2]. International guidelines strongly recommend the use of the synthetic lysine analog-tranexamic acid (TXA) in the early care of bleeding trauma patients at risk of significant hemorrhage [1, 5]. Thus, off-label early administration of this anti-fibrinolytic agent [1] has become a significant component of major hemorrhage protocols [12], but its infusion after 3 h from injury has been associated with the potential occurrence of adverse effects such as nausea, diarrhea, and stomach ache [1, 13]. Ideally, initial administration of TXA should be considered in the pre-hospital phase where possible, as evidenced by the decreasing survival benefit over time [14]. Current evidence suggests that early TXA hemostatic administration may improve outcomes in trauma patients both with hemorrhagic shock and intracranial bleeding [15]. Moreover, in cases of uncontrollable hemorrhage and coagulopathy, the off-label use of TXA has been suggested [1, 16]. While recombinant activated coagulation factor VII (rFVIIa) has been rarely investigated and used in the pre-hospital arena [17], TXA was tested by several published studies [18].

This systematic review aimed to assess the effectiveness and safety of systemic hemostatic agents as adjunctive treatment measures initiated in the pre-hospital setting and then continued in the in-hospital setting in patients with major trauma and hemorrhage or with TBI.

## Methods

A systematic review was conducted to support the major trauma integrated management guideline panel of the Italian National Institute of Health (*Istituto Superiore di Sanità*, *Sistema Nazionale Linee Guida*) in formulating recommendations. Specifically, following the Grading of Recommendations Assessment, Development and Evaluation (GRADE)-ADOLOPMENT approach for guideline creation [19] adopted by the *Istituto Superiore di Sanità* methodological manual, the panel members decided to follow a structured and systematic adaptation and updating process of the recommended use of systemic hemostatic agents initiated in the pre-hospital setting from the National Institute for Health and Clinical Excellence (NICE) on major trauma (clinical guideline, NG39) [20]. The clinical question addressed in this systematic review was, “Is the use of systemic hemostatic agents clinically effective in improving outcomes in patients with confirmed or suspected hemorrhage in major trauma or with acute TBI?”.

### Study design

The systematic review followed the Preferred Reporting Items for Systematic Reviews and Meta-analyses (PRISMA) reporting guideline (Supplemental Material 1) [21]. The study protocol is available at the following link: https://osf.io/cmdqk/.

### Search strategy

Two professional librarians searched the Embase, PubMed (Medline), and the Cochrane Library databases for randomized controlled trials (RCTs) published up to October 12, 2021, that were related to the use of systemic hemostatic agents introduced in the pre-hospital environment among patients with confirmed or suspected hemorrhage or with acute TBI. Following the GRADE-ADOLOPMENT development process [19], the search strategy included in the high-quality NICE guideline on major trauma from 2015 [20] has been updated.

Full details of the search strategy are reported in Supplemental Material 2.

### Study selection

Two independent authors (A.B. and G.P.) completed reference screening and study selection using the following pre-defined inclusion criteria: (1) *population*: children, young people, or adults who have experienced a suspected hemorrhage or TBI following a traumatic incident; (2) *intervention*: administration of a systemic hemostatic agent, (e.g., rFVIIa, TXA, fibrinogen concentrate, prothrombin complex concentrate, or another anti-fibrinolytic agent); (3) *comparison*: no treatment, placebo, standard care (e.g., crystalloids, blood components), or any other hemostatic agent; and (iv) *setting:* systemic hemostatic treatment initiated in the pre-hospital setting. Only RCTs were considered eligible for inclusion; case reports, editorials, and letters were excluded from the search.

When multiple publications for the same trial reported different outcomes or follow-up data, they were counted as a single publication. Discrepancies between reviewers were resolved by consulting a third author (O.C.).

### Types of outcome measures and follow-up assessment

The primary outcome was the overall mortality (at 24 or 48 h or 1 month), while the secondary outcomes were cause-specific mortality at 1 month (e.g., multiple organ failure [MOF], head injury, hemorrhage), health-related quality of life; any adverse effect (e.g., MOF); blood product use; mortality at 12 months, hemorrhage expansion; and patient-reported outcomes.

### Data extraction

Two independent authors (A.B. and G.P.) extracted data on publication year, country, and characteristics of the study population (e.g., age, Injury Severity Score [ISS], systolic blood pressure, heart rate, Glasgow Coma Scale, type of injury [blunt %], type of systemic hemostatic agents, and outcome data) using a standardized data-collection form in a spreadsheet format (Microsoft Excel, Redmond, WA, USA). Authors were contacted if the reported data were insufficient or unclear.

### Statistical analysis

General characteristics were descriptively synthesized. When sufficient outcome data were available, cumulative analyses were performed. Treatment effects provided an estimate of the risk ratio (RR) for dichotomized outcomes and a mean difference (MD) or standardized mean difference (SMD) value when different outcome measurements were present for continuous outcomes, along with 95% confidence intervals (CI). Heterogeneity between study-specific estimates was tested using chi-squared statistics and measured with the I^2^ index and the Cochran’s Q test [22, 23]. A pooled estimate was obtained by fitting the DerSimonian and Laird random-effects model [24] when several studies were combined; conversely, a fixed-effects model was applied. Subgroup analyses were planned for (1) type of population (e.g., subjects with significant hemorrhage and subjects with TBI); (2) type of injury (e.g., blunt trauma, penetrating trauma); and (3) doses of systemic hemostatic agent when available. All tests were considered significant statistically for *p* < 0.05. For the primary outcome (i.e., mortality) the clinical relevance was assessed with the minimal important differences (MIDs) taken as risk ratios (RRs) of 0.75 and 1.25. For instance, the RR of 0.75 of mortality is taken as the line denoting the boundary between no clinically important effect and a clinically significant benefit, whilst the RR of 1.25 is taken as the line denoting the boundary between no clinically important effect and a clinically significant harm [20]. The analyses were performed using Review Manager Version 5.4 (Cochrane Collaboration, London, UK).

### Risk-of-bias assessment (internal validity)

The Cochrane risk-of-bias tool was used to evaluate the internal validity of the included RCTs. The quality of the evidence was assessed using the GRADE methodology [25] (Supplemental Material 3).

## Results

### Study selection

**Fig. 1:**
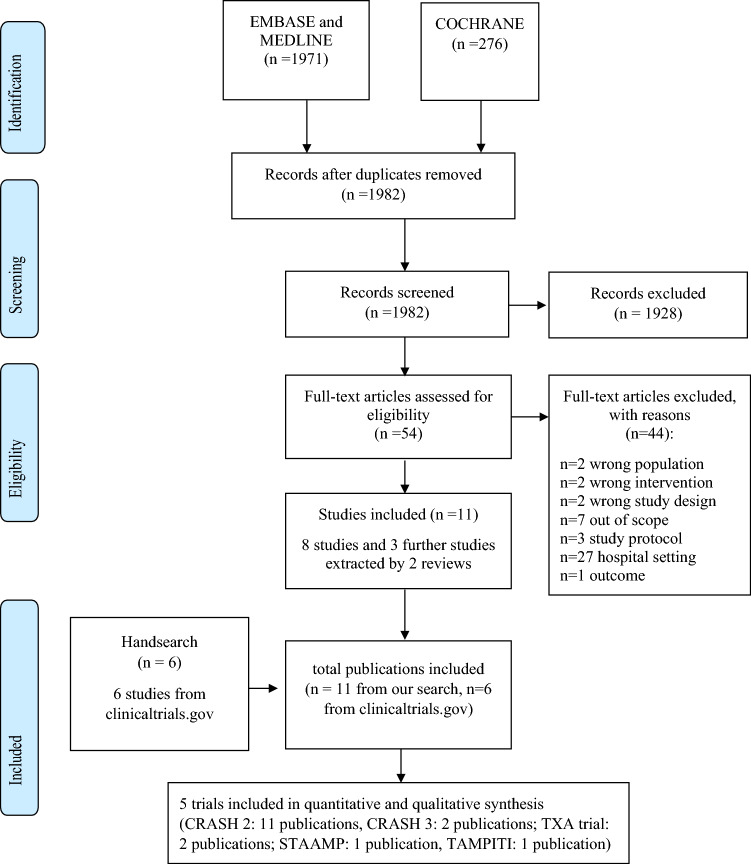
Flow diagram of study selection

Overall, 1982 records were identified by updating the search. Ten references met the study eligibility criteria, including eight primary studies (i.e., RCTs) [2, 26–31] and two systematic reviews [32, 33], out of which three studies were extracted [4, 34]. One study [5] was already included in the NICE guideline. In addition, six references were sourced from ClinicalTrials.gov [35–40]. After performing an overlapping evaluation between studies included in the retrieved systematic reviews and primary studies from the updated search strategy, ongoing trials, and the one [5] already included in the NICE guideline, 17 studies related to seven RCTs were included (Fig. 1). In Supplemental Material 2, all included publications related to the five RCTs are listed, namely CRASH-2 [4, 5, 27, 29, 34–40], CRASH-3 [2, 26], the TXA trial [28, 41], STAAMP [30], and TAMPITI [31]. No eligible study was found on the use of fibrinogen concentrates, recombinant activated coagulation factor VII, prothrombin complex concentrates, or other anti-fibrinolytic agents in the pre-hospital setting.

### General characteristics

Overall, five RCTs assessed the comparison of TXA and placebo (Supplemental Material 2). The median ISS across studies ranged from a minimum of 12 (interquartile range [IQR] = 5–22) to a maximum of 38.7 (IQR = 25–52.4). Blunt trauma was the most representative feature of patients across studies. In particular, TXA versus placebo was investigated in patients with significant hemorrhage [5, 31, 36, 40] or who were at risk of hemorrhage [30], in patients with isolated TBI [2, 26, 41], and in those with brain injury and significant hemorrhage [4, 34]. The general and clinical characteristics of patients are presented in Table 1.

**Table 1:** General characteristics

Trial, name and publication period	Countries	Type of population (ISS, SBP, HR, GCS) Blunt or penetrating trauma (%) TBI patients (%)	Intervention (*n*) Comparison (*n*)	Funding
CRASH-2, multi-center RCT Studies published from 2011 to 2019	274 hospitals in 40 countries (Albania, Argentina, Australia, Bangladesh, Belgium, Cameroon, Canada, China, Colombia, Cuba, Czech Republic, Ecuador, Egypt, El Salvador, Georgia, Ghana, India, Indonesia, Iran, Iraq, Italy, Jamaica, Japan, Kenya, Malaysia, Mexico, Nigeria, Peru, Saudi Arabia, Serbia, Singapore, Slovakia, South Africa, Spain, Sri Lanka, Tanzania, Thailand, Tunisia, UK, Zambia)	Adult trauma patients with significant hemorrhage (SBP < 90 mmHg, HR > 110 beats per minute or both; ISS not reported; GCS was detected in TXA and placebo (ranged as mild: 17.8% vs. 18.2%, moderate: 13.4% both, severe: 68.7% vs. 68.3%; while in TBI patients, the median GCS was 10.5) Blunt trauma: 67.6% Subgroup of patients had a TBI: 1.34%	TXA: 1 g in the pre-hospital setting followed by a 1 g maintenance infusion initiated on hospital arrival and infused over 8 h (*n* = 10,093) Placebo (*n* = 10,114)	Government No-profit Industry
CRASH-3, multi-center RCT Studies published from 2019 to 2021	175 hospitals in 29 countries (Pakistan, UK, Malaysia, Georgia, Spain, Nigeria, Colombia, Nepal, Albania, Japan, United Arab Emirates, Myanmar, Cameroon, Afghanistan, Mexico, Italy, Iraq, Cambodia, Zambia, Romania, El Salvador, Egypt, Slovenia, Ireland, Papua New Guinea, Canada, Jamaica, Indonesia, Kenya)	Patients with TBI (GCS ≤ 12), treated within 3 h from injury. Adults with TBI, GCS score of 12 or lower or any intracranial bleeding on CT scan, and no major extracranial bleeding. The time window for eligibility was originally 8 h, but in 2016 the protocol was changed to limit recruitment to patients within 3 h of injury ISS = not reported Blunt trauma not reported	TXA: 1 g in the pre-hospital setting followed by a 1 g maintenance infusion initiated on hospital arrival and infused over 8 h (*n* = 4649) Placebo (*n* = 4553)	Government No-profit
STAAMP, multi-center RCT Study published in 2020	1 of 4 US level 1 trauma centers	Injured patients at risk for hemorrhage who experienced at least one episode of hypotension (SBP ≤ 90 mmHg) or tachycardia (heart rate ≥ 110 beats per minute) Patients had a median ISS of 12 (interquartile range [IQR], 5–22) specifically among the TXA group (13) The median (IQR) pre-hospital heart rate and SBP were detected among TXA (118 [112–117] and 123 [88–143]) and placebo (117 [112–124] and 126 [87–148]) arms The number of patients with GCS < 8 in the TXA and placebo group were 89 (19.9%) and 107 (23.5%), respectively Blunt trauma mechanisms among 85.3% in placebo group vs. 83% in TXA group Penetrating trauma mechanisms among 15.4% in placebo group vs. 17.4% in TXA group Severe TBI among 17% in the placebo group and 20% in the TXA group	TXA: Phase A (pre-hospital: 1 g TXA in 10 mL solution or 10 mL sterile water placebo and infused for 10 min) Phase B (in-hospital: 1 g TXA in 10 mL solution or 10 mL placebo (sterile water) and infused for 10 min) Phase C (in-hospital: 1 g TXA in 10 mL solution or 10 mL placebo and infused for 8 h) The abbreviated dosing regimen was 1 g TXA bolus (phase A), placebo bolus (phase B), and placebo infusion (phase C). The standard dosing regimen was 1 g TXA bolus (phase A), placebo bolus (phase B), and 1 g TXA infusion (phase C). The repeat bolus dosing regimen was 1 g TXA bolus (phase A), 1 g TXA bolus (phase B), and 1 g TXA infusion (phase C) (*n* = 447) Placebo: placebo bolus (phase A), placebo bolus (phase B), and placebo infusion (phase C) (*n* = 456)	Government No-profit
TAMPITI, single-center RCT Study published in 2020	USA. Washington University in St. Louis, a tertiary care center	Patients who sustained a traumatic injury which required them to receive at least one unit of red blood cells (RBC) or required an emergency operation were included The median (IQR) ISS and GCS were detected, respectively, in the TXA (2 g: 22 [16–34] for the ISS and 15 [11–15] for the GCS; 4 g: 19 [14–29] for the ISS and 15 [14, 15] for the GCS) and placebo (19.5 [14–34] for the ISS and 15 [12–15] for the GCS) groups. The median (IQR) HR and SBP was found in the TXA (2 g: 102 [90–128] for the ISS and 119 [90–142] for the SBP; 4 g: 100.5 [80–117] for the HR and 118 [98–135] for the SBP) and placebo (97 [83–120] for the HR and 120 [98–141] for the SBP) arm Penetrating injury was found in 77.6% and 78% of patients treated with 2 g and 4 g TXA, respectively, while it was found in 82% of patients in the placebo arm	TXA 2 g TXA, or 4 g TXA in 40 mL normal saline intravenously over 10 min within 2 h of initial injury (*n* = 49 for 2 g TXA; * n* = 50 for 4 g TXA) Placebo: (*n* = 50)	Government No-profit
TXA trial, multi-center RCT Studies published during 2020	20 trauma centers and 39 emergency medical services agencies in the USA and Canada	Patients with blunt or penetrating traumatic mechanism consistent with TBI, pre-hospital GCS score ≤ 12 at any time prior to randomization and administration of sedative and/or paralytic agents, pre-hospital SBP ≥ 90 mmHg prior to randomization, age ≥ 15 (or estimated weight > 50 kg if age is unknown) and Emergency Medicine System transport to a participating trauma center ISS [mean (IQR)] = 17 [8–27] Blunt trauma: 67%	TXA group 1: 1 g in the pre-hospital setting followed by a 1 g maintenance infusion initiated on hospital arrival and infused over 8 h TXA group 2: 2 g intravenous TXA in the pre-hospital setting followed by a placebo maintenance infusion initiated on hospital arrival and infused over 8 h (*n* = 657) Placebo (*n* = 309)	Government No-profit

*GCS* Glasgow Coma Scale, *HR* heart rate, *ISS* injury severity score, *SBP* systolic blood pressure, *TBI* traumatic brain injury, *TXA* tranexamic acid

### Overall mortality

All included RCTs (*n* = 5) reported the overall mortality data. The CRASH-2 and STAAMP trials showed mortality at 24 h, while five RCTs (CRASH-2, CRASH-3, STAAMP, TAMPITI, and the TXA trial) evaluated overall mortality at 1 month.

### TXA versus placebo

**Fig. 2:**
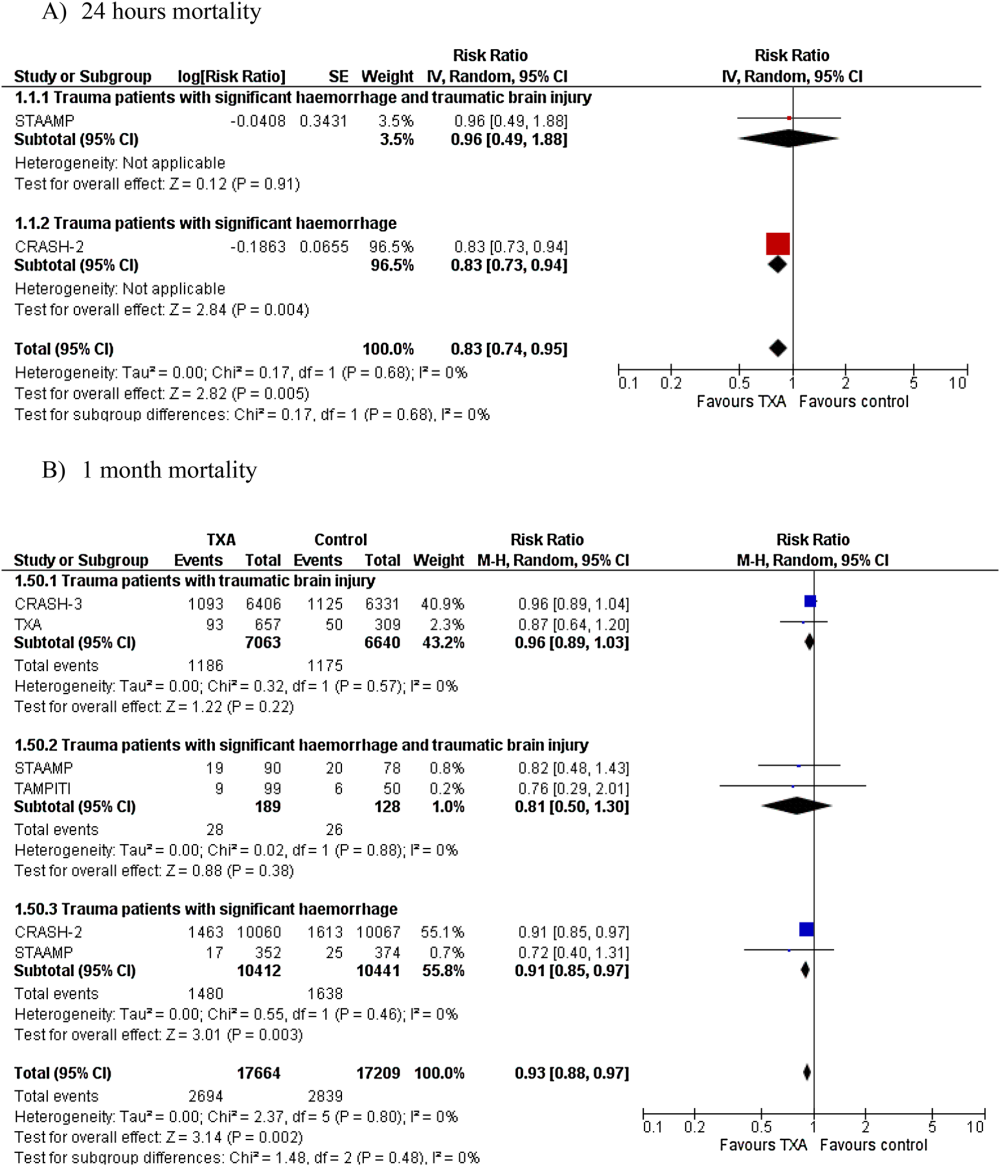
Risk ratio for overall mortality (1 month) of TXA vs. placebo

A statistically significant difference including clinical relevance between TXA and placebo for overall mortality at 24 h was observed (RR = 0.83, 95% CI = 0.74–0.95; two studies, 21,030 patients; Fig. 2A), whereas a statistically but not clinically significant reduction was found at 1 month in the TXA group (RR = 0.93, 95% CI = 0.88–0.97; five studies, 34,873 patients, Fig. 2B). The absolute effects with TXA shown 8 and 12 fewer deaths per 1.000 at 24 h and 1 month, respectively.

In the subgroup analysis for the type of population (Fig. S1 in Supplemental Material 4), TXA did not reduce the overall mortality at 1 month in patients with TBI (RR = 0.96, 95% CI = 0.89–1.03; two studies, 13,703 participants) or those with TBI and significant hemorrhage (RR = 0.72; 95% CI = 0.49–1.05, three studies, 587 participants) compared to placebo; however, a reduction was registered among trauma patients with significant hemorrhage (RR = 0.91, 95% CI = 0.85–0.97; two studies, 20,853 participants; Table S2 in Supplemental Material 4).

### Secondary outcome

Descriptive tables and quantitative analyses for the following outcomes are shown in Supplemental Material 4.

- Cause-specific mortality: Bleeding death at 1 month was significantly reduced in patients who received TXA compared to placebo, whereas no differences between the groups at 1 month were detected for MOF, head injury, pulmonary embolism, and sepsis (Table S3 and Figs. S3–S5 in Supplemental Material 4).
- Health-related quality of life: There was no clear effect of TXA on disability among survivors, as evaluated by the Disability Rating Scale [42] in two RCTs [2, 28] (Fig. S6 in Supplemental Material 4).
- Adverse effects: Reported data on sepsis, pulmonary embolism, myocardial infarction or stroke, renal failure, seizures, gastrointestinal bleeding, acute respiratory distress syndrome (ARDS), MOF, and vascular occlusive events did not demonstrate an increased risk with the use of TXA compared to placebo (Table S4 and Figs. S7–S16 in Supplemental Material 4).
- Blood product use: There was no difference in blood, platelet, or plasma transfusions between groups (Table S5 in Supplemental Material 4).
- Other secondary outcomes: None of the RCTs reported the mortality at 12 months or patient-reported outcomes, and no significant reduction was detected regarding hemorrhage expansion (Table S6 in Supplemental Material 4).

The above results suggest a higher safety profile of TXA therapy without a significant difference in complications between the two groups.

### Internal validity and certainty of evidence

Five RCTs (CRASH-2, CRASH-3, STAAMP, TAMPITI, and TXA trial) were judged to have good methodological quality (Supplemental Material 3). The certainty of evidence across the RCTs ranged from very low to high quality (Table 2).

**Table 2:** Summary of Findings for the Overall Risk of Mortality in the TXA vs. Placebo

Outcomes	No of participants (studies) Follow-up	Certainty of the evidence (GRADE)	Relative effect (95% CI)	Anticipated absolute effects	
				Risk with placebo	Risk difference with TXA
Mortality 24 h	21,030 (2 RCTs)	⨁⨁⨁⨁ High	**RR 0.83** (0.74 to 0.95)	48 per 1.000	**8 fewer per 1.000** (13 fewer to 2 fewer)
Mortality 1 month	34,882 (5 RCTs)	⨁⨁⨁⨁ High	**RR 0.93** (0.88 to 0.97)	165 per 1.000	**12 fewer per 1.000** (20 fewer to 5 fewer)
*The risk in the intervention group (and its 95% confidence interval) is based on the assumed risk in the comparison group and the relative effect of the intervention (and its 95% CI) CI: confidence interval; MD: mean difference; RR: risk ratio					
GRADE Working Group grades of evidence High certainty: we are very confident that the true effect lies close to that of the estimate of the effect Moderate certainty: we are moderately confident in the effect estimate: the true effect is likely to be close to the estimate of the effect, but there is a possibility that it is substantially different Low certainty: our confidence in the effect estimate is limited: the true effect may be substantially different from the estimate of the effect Very low certainty: we have very little confidence in the effect estimate: the true effect is likely to be substantially different from the estimate of effect					

## Discussion

To our knowledge, this is the first comprehensive systematic review with a meta-analysis assessing the certainty of evidence by the GRADE approach to prove the efficacy of systemic hemostatic agents as an adjunctive treatment initiated in the pre-hospital setting among traumatic patients with (1) hemorrhage, (2) TBI, or (3) hemorrhage and TBI. With a high certainty of evidence, TXA is associated with a lower risk of mortality of 17% at 24 h (RR = 0.83, 95% CI = 0.74–0.95) and 7% at one month (RR = 0.93, 95% CI 0.88–0.97) when compared to placebo. Although the clinical relevance could be questionable, the anticipated absolute effects at 24 h and 1 month anyway shown a reduction of mortality of 8 and 12 averted deaths per 1000 which may be relevant in a life threatening conditions (i.e., major trauma). Considering the subgroup of severe TBI at 24 h and one month, both failed to find any difference between groups.

Difference between patients with severe hemorrhage and TBI may be explained by the timing of anti-fibrinolytic administration and the severity of TBI [2, 26]. TBI, commonly characterized by intracranial hemorrhage, is particularly concerning in this context because it can progress or gradually worsen after hospitalization [43]. One-third of patients affected by severe TBI may develop coagulopathy because of the release of brain phospholipids and tissue factors [44]. However, a recent meta-analysis did not show a strong prevalence of coagulopathy in TBI patients compared to those with injuries in other areas of the body or multiple injuries with TBI [45]. Especially in the early stages [46], TXA administration tends to reduce hemorrhage expansion and mortality caused by bleeding in TBI patients [34]; nevertheless, those affected by intracranial bleeding and neuropathological abnormalities did not show significant benefits of anti-fibrinolytic treatment [26]. Within 24 h of injury deaths are more likely to occur because of excessive bleeding [2].

We can use 24-h mortality as a proxy of the overall mortality effect [6], since the ideal endpoint in literature is considered as mortality within the first 6 h after injury [14, 47], in fact the CRASH-2, which represents the most contribution (96.5% of weight of meta-analysis), administered the TXA within 3 h from injury. The effect of systemic hemostatic agents depends on the time between injury and the onset of treatment, which suggests there is a protective effect of these medications on early deaths related to hemorrhage [48].

The treatment benefit is time-dependent, the benefits of TXA therapy seen in the first 3 h may be explained by the increasing levels of plasminogen activator inhibitor-1 and a reduction in fibrinolysis. In CRASH-2, when TXA was administered within 3 h of injury, the risk of death due to bleeding was reduced [29]. However, in the late phase of TXA administration, adverse effects, especially thrombotic disseminated intravascular coagulation, was likely to increase, along with uncontrolled bleeding [29] and a higher risk of mortality [49].

Therefore, its administration may be contraindicated [36]. However, we found no differences between TXA and placebo in terms of adverse effects. In addition, many guidelines have focused on TXA administration in severely injured or bleeding patients [20]. The British Committee for Standards in Hematology recommends the administration of TXA in adult trauma patients with, or who are at risk of, bleeding as early as possible after injury [50], and the STOP the Bleeding Campaign in Europe also recommends its use in bleeding trauma patients within 3 h after an injury and those “en route” to the hospital [51]. On the basis of existing evidence, TXA was also included on the World Health Organization’s list of essential medicine for the reduction of death among adult patients with trauma and a significant risk of ongoing hemorrhage [52].

Our results are in agreement with those in the current scientific literature. Specifically, meta-analyses and systematic reviews conducted in the pre-hospital or the hospital setting found that TXA reduces the risk of death from all causes by about 20% [44, 53, 54] and less hemorrhagic expansion [44] among TBI patients [32, 46]; yet some results were discrepant with respect to functional status [32, 44, 54, 55]. No evidence was detected for other adverse events [32, 44, 46, 55], although high-quality RCTs reported reduced vascular occlusive events [44]. However, confounders may have influenced the results, such as blood transfusions realized prior to TXA treatment, although this mechanism is still unknown [53].

This study has some limitations. First, survival bias may be present when patients die before the administration of treatment; thus, the results might not be generalizable across the trauma population [56]. Second, cause-specific mortality can be affected by a subjective and misclassified evaluation (e.g., an in-hospital death for hemorrhage may be caused by hemorrhage and severe TBI [6]). Third, clinical conditions (e.g., cardiac arrest) may have strongly influenced the treatment indication. Statistical analyses may not have taken into account the time-varying treatment effect by assuming uniform effects over time, nor adequately considered risk factors [56], or the study outcomes (e.g., reduction of mortality) [39]. Thus, appropriate strategies may reduce these type of biases [56]. Some studies also did not specify the type of trauma (blunt or penetrating) and did not categorize patients based on their trauma severity, while others performed secondary analyses or re-analyses (e.g., restriction to TBI patients). These factors may have limited the subgroup analysis. Fourth, inclusion criteria were narrow, aiming to balance pros and cons. We considered eligible all RCTs that investigated several interventions delivered in any setting (civilian and military). Thus, although we were able to offer the highest level of evidence (RCTs with high certainty of evidence) achieving more consistency in the interpretation of findings, this approach limited the results as we did not find studies in military setting or studies involving fibrinogen concentrates, prothrombin complex concentrate, or other anti-fibrinolytic agents. The inclusion observational evidence (e.g., non-randomized interventional studies) a different pragmatic approach have been found. Early patient randomization may not have prevented the performance of stratified analysis on the anatomical location of bleeding or other injury [36]. Moreover, difficult diagnoses of traumatic hemorrhages might have reduced the statistical power analysis focused on the anti-fibrinolytic effect on bleeding mortality or the need for blood transfusions [5]. Fifth, heterogeneity was found in reporting the average time from injury to anti-fibrinolytic administration, as well as the time of data collection regarding outcome. Last, the complexity of the trauma cohorts and different choices made by trauma teams in their management across various countries may have affected the overall analyses [30].

## Conclusion

Balancing benefits and harms, tranexamic acid initiated in the pre-hospital setting can be used for patients experiencing major trauma with significant hemorrhage. Considering the ideal endpoint in literature is the administration of TXA within 6 h from injury, TXA statistically and clinically reduces mortality rate in trauma patients at 24 h. This result is still statistically significant at 1 month. These results are mainly dragged by the subgroup of patients with significant hemorrhage because in TBI patients subgroup there are no difference between TXA and control. Little-to-no difference in terms of adverse effects was found when comparing TXA and placebo. Further research is needed to investigate the role of other systemic hemostatic agents in the pre-hospital settings.

Supplemental Material 1. PRISMA 2009 Checklist.

Supplemental Material 2. Search strategy and study references.

Supplemental Material 3. Internal Validity and Certainty of the evidence.

Supplemental Material 4. Additional results: primary and secondary outcomes.
